# High protein intake is associated with low plasma NAD^+^ levels in a healthy human cohort

**DOI:** 10.1371/journal.pone.0201968

**Published:** 2018-08-16

**Authors:** Neda Seyedsadjadi, Jade Berg, Ayse A. Bilgin, Nady Braidy, Chris Salonikas, Ross Grant

**Affiliations:** 1 School of Medical Sciences, Faculty of Medicine, University of New South Wales, Sydney, NSW, Australia; 2 Australasian Research Institute, Sydney Adventist Hospital, Sydney, NSW, Australia; 3 Department of Statistics, Macquarie University, Sydney, NSW, Australia; 4 Centre for Healthy Brain Ageing, School of Psychiatry, Faculty of Medicine, University of New South Wales, Sydney, Australia; 5 Sydney Adventist Hospital Clinical School, University of Sydney, Sydney, NSW, Australia; Laurentian University, CANADA

## Abstract

High protein intake and reduced levels of the essential pyridine nucleotide nicotinamide adenine dinucleotide (NAD^+^) have both been independently associated with promotion of the ageing phenotype. However, it has not yet been shown whether these two independent observations are biochemically linked. To investigate this possibility, we used a cross-sectional study design of 100 apparently healthy middle-aged males (n = 48) and females, in which we assessed average dietary intakes of multiple components using a validated questionnaire. We also analysed fasting blood levels of urea, NAD^+^ and its metabolites, and inflammation-linked biomarkers, including interleukin-6 (IL-6), Kynurenine (Kyn), and Tryptophan (Trp). One-way ANOVA and ANCOVA were then performed for statistical analysis. Our results have shown for the first time that plasma levels of NAD^+^ and Total NAD(H) were lower with increasing protein intake (F (2, 92) = 4.61, *P* = 0.012; F (2, 92) = 4.55, *P* = 0.013, respectively). The associated decrease in NAD^+^ and NAD(H) levels was even stronger with increasing plasma levels of the protein breakdown product urea (F (2, 93) = 25.11, *P*≤0.001; F (2, 93) = 21.10, *P*≤0.001). These associations were all independent of age, gender and energy intake. However, no significant association was observed between protein intake or plasma urea, and plasma levels of NAD^+^ metabolites. We also observed that plasma levels of the inflammatory cytokine IL-6, and both Kyn, and Trp, but not the Kyn/Trp ratio were higher with increasing plasma urea levels (F (2, 94) = 3.30, *P* = 0.041; F (2, 95) = 7.41, *P*≤0.001; F (2, 96) = 4.23, *P* = 0.017, respectively). These associations were dependent on eGFR and energy intake, except for the urea and Trp association that was independent of all. In conclusion, we report for the first time, a novel association between protein intake, its metabolism, and plasma NAD^+^ levels with a possible link to inflammation. These findings provide further insight into how protein restriction may contribute to the anti-ageing process observed in several studies.

## Introduction

Numerous studies have shown that diet and nutritional status play an important role in the development of a range of age-related degenerative diseases including cardiovascular disease, diabetes, dementia and several cancers [1, 2]. As a consequence, there is growing awareness that this knowledge may be harnessed to not only prevent and ameliorate many diseases, but significantly extend lifespan. It is well established that simply reducing calorie intake dramatically reduces the risk of some common diseases and significantly increases the average lifespan [3, 4]. However, it is still not clear whether these benefits are due to the reduction in calorie intake or the associated decrease in specific nutritional components such as proteins [5, 6].

In recent years, several studies using various animal models have reported that protein restriction can specifically slow the ageing phenotype and extend lifespan [7, 8]. In a review of the literature, Pamplona and Barja stated that protein restriction (40–85% of standard chow) independently increased lifespan in rodents by about 20% [8]. In humans, a recent study of a middle-aged population revealed that subjects with the highest protein intake (more than 20% of calories from protein) had a 75% increase in all-cause mortality and a four-fold increase in diabetes and cancer mortality [9].

A suggested rationale for these observations is that protein restriction can affect major metabolic pathways implicated in the regulation of lifespan such as the mechanistic target of rapamycin (mTOR) [10], insulin [11], insulin like growth factor 1 (IGF-1) [9] and fibroblast growth factor 21 (FGF21) [11]. However, the mechanism(s) driving these associations are yet to be fully understood.

Nicotinamide adenine dinucleotide (NAD^+^) is a ubiquitous pyridine nucleotide which plays a critical role in cell viability and ageing by taking part in a range of vital cellular processes, including energy production [12], DNA repair [13] and epigenetic signalling [14]. In addition to its well-known role as an essential coenzyme in ATP production and over four hundred different redox reactions [12], NAD^+^ has also been shown to act as the primary substrate for three important classes of enzymes, poly (ADP ribose) polymerases (PARPs), NAD^+^ glycohydrolases and sirtuins (SIRTs). PARPs are a family of proteins involved in a number of cellular processes including DNA repair, genomic stability, and programmed cell death [13]. Glycohydrolases such as the cluster of differentiation 38 (CD38), catabolise NAD^+^ to produce nicotinamide and cyclic adenosine diphosphoribose (cADPR) mediating both intracellular calcium signalling and lymphocyte chemotaxis [14, 15]. NAD^+^ is also an essential substrate for the class III protein deacetylases, sirtuins (SIRTs 1–7) [16, 17], which are involved in many cellular processes including the epigenetic regulation of genes involved in cellular senescence and ageing [18]. Importantly, our laboratory [19, 20], and others [21] have observed a significant reduction in NAD^+^ levels with age in both animal and human tissues. However, it is yet to be determined if this decrease in NAD^+^ is primarily causal or secondary to changes in PARP, glycohydrolase and SIRT activities with age. As both high protein intake and reduced cellular NAD^+^ levels have been associated with accelerated ageing, the question arises: Could protein intake affect NAD^+^ levels? To help answer this question, we investigated possible associations between protein intake, plasma urea levels, and plasma NAD^+^ and NAD^+^ metabolite levels in an apparently healthy middle-aged population. We provide evidence for the first time of a relatively robust association between protein intake and NAD^+^ metabolism and propose a theoretical mechanism that may help explain these observations.

## Materials and methods

### Participants

This cross-sectional study included 100 participants (48 males and 52 females) aged between 40 and 75 years, in general good health. Participant recruitment was conducted at the Sydney Adventist hospital, and the University of New South Wales Sydney via advertisements in the hospital/university intranet. Volunteers were excluded if they had any current microbial infection, had suffered from any form of neurodegenerative disease, a medically diagnosed liver or kidney disorder, or inflammatory bowel disease, had been diagnosed with cancer or been treated for cancer, had major surgery in the past two years, had taken any diabetic, or thyroid medications, had taken any antibiotics, or anti-inflammatory medications, illicit drugs, supplements or any complementary medicines in the last two weeks prior to testing. After obtaining a written informed consent, eligible participants were asked to complete a dietary questionnaire for the assessment of their dietary intakes (including protein). Blood collection was performed in a fasted state (about 12 hours) on the same day as the completion of the questionnaire. Ethical approval of the study was obtained from the Adventist HealthCare Limited Human Research Ethics Committee, Sydney Adventist Hospital, Australia (HREC number: 2013–022).

### Protein and energy intake assessment

Protein and energy intake was assessed by the validated 74-item Cancer Council Victoria Dietary Questionnaire for Epidemiological Studies Version 2 (DQES v2) that evaluates dietary intakes of multiple components over the past twelve months [22].

### Biochemical analysis

The measurement of plasma urea and calculation of eGFR were performed by the Sydney Adventist Hospital pathology laboratory using methods well established for clinical laboratories. Briefly, fasting plasma urea levels were determined by the enzymatic method on a Roche/Hitachi cobas c system. Estimated Glomerular filtration rate (eGFR) was calculated using the simplified Modification of Diet in Renal Disease study equation: GFR (ml/min/1.73m^2^) = 186 × (serum creatinine level [mg/dl])-1.154 × (age)-0.203× [0.742, if female] × [1.212, if black] [23, 24].

Plasma levels of NAD^+^ and its metabolites [NADP^+^, cyclic ADP ribose (cADPR), nicotinamide (NAM), N-methylnicotinamide (MeNAM)] were measured by liquid chromatography coupled to tandem mass spectrometry (LC/MS/MS), as previously described [25]. LC/MS/MS was carried out using a UPLC-MSD assembly consisting of an Accela UPLC pump, Accela AS injector, and a TSQ Vantage bench-top mass spectrometer (ThermoFisher Scientific, Waltham, US).

Plasma interleukin-6 (IL-6) levels were quantitated using the MILLIPLEX® MAP Human High Sensitivity T Cell Magnetic Bead Panel immunoassay (Merck KGaA, Darmstadt, Germany).

Plasma Kynurenine (Kyn) and Tryptophan (Trp) levels were measured by high-performance liquid chromatography (Shimadzu LC-10AVP system, equipped with SPD- 10A UV detector, Kyoto, Japan), as previously described [26].

### Statistical analysis

SPSS version 23 for Windows was used for Statistical analysis [27]. Data is presented as means ± standard deviations unless otherwise stated. Both the Shapiro-Wilk, Kolmogorov-Smirnov and histogram analysis were used to check normality of the variables. If the normality tests for the variables were significant then base-10 log-transformed means or square roots were used where needed. Outliers were defined as values above or below mean +/- three standard deviations. After applying this rule and also after checking graphical displays, one outlier was removed for variables, protein intake, energy intake, urea, NADH, NAD^+^/NADH, IL-6, Kyn, and Kyn/Trp ratio, and three outliers were removed for variables NAM and MeNAM. For the tertile categorization of protein intake and plasma urea, cut-points were set for three equal groups and then the new categorical variables (tertiles) were defined based on the related cut-point values. One-way ANOVA followed by Tukey’s post hoc test were used to determine statistically significant differences between the tertiles. The Levene's Test of Equality was applied to check homogeneity of variances between tertiles. One-way ANCOVA was used for adjustment for possible confounders when analysing differences between tertiles. Bonferroni adjustment was conducted to allow for the multiple comparisons involved. The association between plasma urea and NAD^+^, Total NAD(H) and NAD^+^/NADH, was further analysed using Pearson’s correlation coefficients. *P*-values are provided with test significance set at *P*-value ≤ 0.05.

## Results

The study cohort consisted of 100 subjects (48 were males) with mean age 56.0 ± 8.8 years. The mean protein intake was 79.29 ± 30.34 g/day and the mean plasma urea level was 5.44 ± 1.51. A complete list of cohort characteristics is shown in Table 1.

**Table 1:** Characteristics of the study participants according to plasma urea tertiles.

Characteristic	Total (Mean ± SD)	Plasma urea (mmol/L)			P-value*
		1^st^ tertile (3.78 ± 0.75)	2^nd^ tertile (5.31 ± 0.45)	3^rd^ tertile (7.12 ± 0.83)	
**Age (years)**	56.0 ± 8.8	52.35 ± 8.52	55.86 ± 9.19	59.88 ± 7.12	≤ 0.001
**Protein Intake (g/day)**	79.29 ± 30.34	69.97 ± 23.24	74.61 ± 32.19	94.44 ± 28.56	0.002
**NAD**^**+**^ **(nmol/L)**	23.86 ± 12.53	33.16 ± 12.95	24.02 ± 10.31	15.26 ± 7.09	≤ 0.001
**NADH (nmol/L)**	20.20 ± 6.83	21.41 ± 5.92	19.70 ± 8.34	19.57 ± 6.00	0.284
**Total NAD(H) (nmol/L)**	44.36 ± 14.57	45.57 ± 13.62	44.61 ± 13.30	34.83 ± 9.67	≤ 0.001
**NAD**^**+**^**/NADH**	1.27 ± 0.80	1.68 ± 0.82	1.34 ± 0.85	0.83 ± 0.45	≤ 0.001
**NADP**^**+**^ **(nmol/L)**	3.70 ± 1.75	3.70 ± 1.69	3.53 ± 1.96	3.89 ± 1.62	0.599
**cADPR (nmol/L)**	9.21 ± 2.30	9.02 ± 2.41	9.39 ± 2.35	9.20 ± 2.21	0.814
**NAM (μmol/L)**	31.99 ± 11.77	33.51 ± 12.91	32.93 ± 11.30	29.66 ± 11.16	0.384
**MeNAM (nmol/L)**	33.90 ± 12.48	35.52 ± 13.68	34.91 ± 11.98	31.44 ± 11.83	0.384
**IL-6 (pmol/L)**	2.67 ± 1.96	2.49 ± 1.56	2.26 ± 2.04	3.31 ± 2.09	0.041
**Kyn (umol/L)**	1.01 ± 0.21	0.93 ± 0.21	0.98 ± 0.19	1.11 ± 0.18	≤ 0.001
**Trp (umol/L)**	46.16 ± 7.39	44.28 ± 8.07	45.08 ± 6.81	49.09 ± 6.58	0.017
**Kyn/Trp ratio**	0.022 ± 0.004	0.021 ± 0.004	0.022 ± 0.004	0.023 ± 0.004	0.322

*Comparison made using one-way ANOVA

NAD^+^, Nicotinamide adenine dinucleotide; NADP^+^, Nicotinamide adenine dinucleotide phosphate; cADPR, Cyclic ADP ribose; NAM, Nicotinamide; MeNAM, *N*-methylnicotinamide; IL-6, Interleukin-6; Kyn, Kynurenine; Trp, Tryptophan.

### Association between protein intake and plasma levels of NAD^+^ and NAD^+^ metabolites

**Fig 1:**
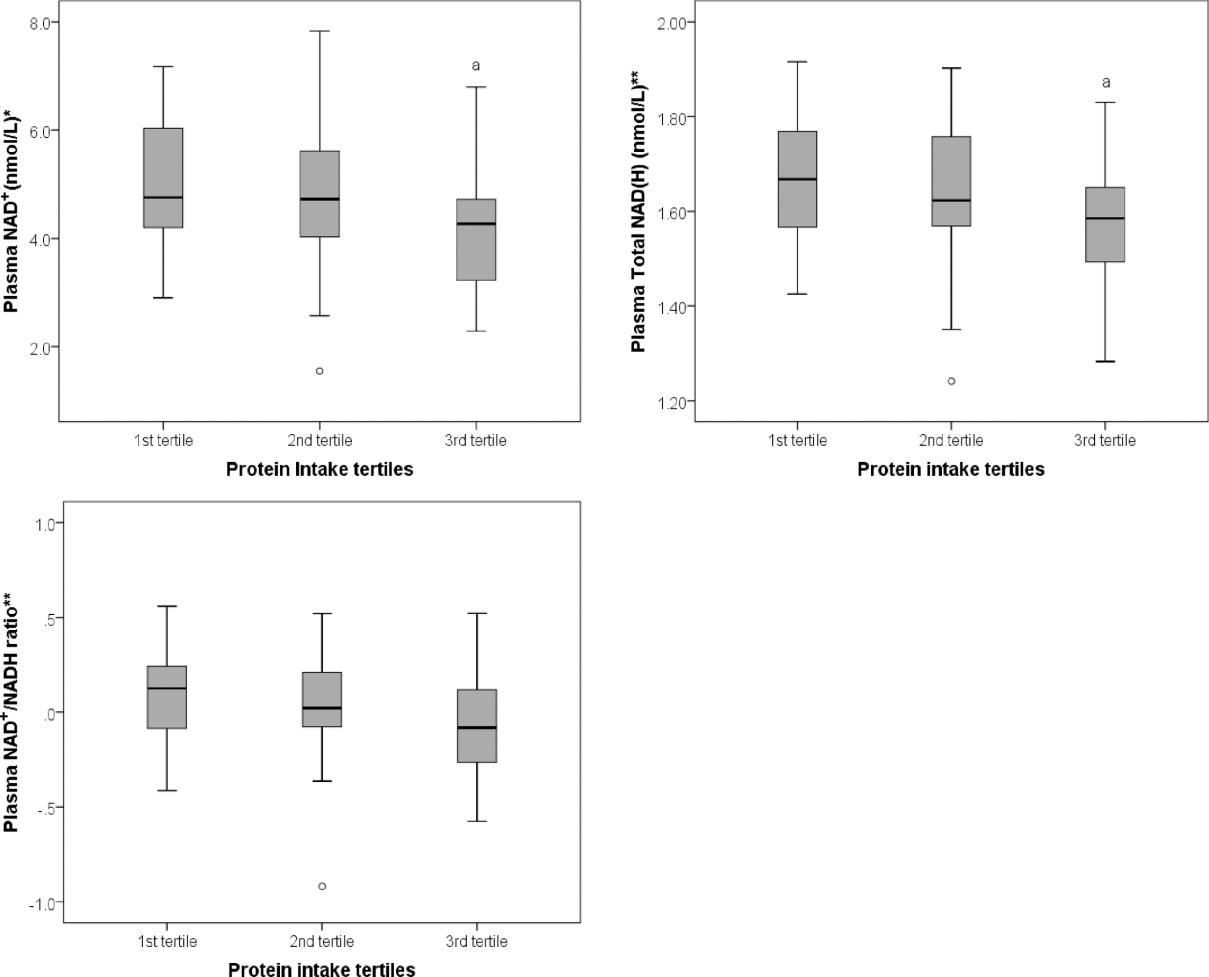
Mean plasma levels of NAD^+^, Total NAD(H), and NAD^+^/NADH ratio across tertiles of plasma urea. *Data represented as square root NAD^+^, **Data represented as base-10 log-transformed Total NAD(H), and NAD^+^/NADH ratio. *a* significantly different compared to 1^st^ tertile.

There was a statistically significant difference in plasma NAD^+^ levels between tertiles of protein intake (F (2, 92) = 4.61, *P* = 0.012) (Fig 1). A Tukey post hoc test revealed that mean plasma NAD^+^ levels were significantly lower in the third tertile compared to the first tertile of protein intake (17.56 ± 1.39 vs. 26.16 ± 1.44, *P* = 0.012). This association remained statistically significant after adjusting for age and gender (F (2, 90) = 4.91, *P* = 0.009).This association remained statistically significant after adjustment for energy intake (F (2, 89) = 3.86, *P* = 0.025), but not after Bonferroni adjustment (P>0.002).

No significant difference was observed in plasma NADH levels between tertiles of protein intake (F (2, 91) = 0.58, *P* = 0.561).

A statistically significant difference in plasma Total NAD(H) (i.e. NAD^+^ + NADH) levels was observed between tertiles of protein intake (F (2, 92) = 4.55, *P* = 0.013) (Fig 1). A Tukey post hoc test revealed that plasma Total NAD(H) levels were significantly lower in the third tertile compared to the first tertile of protein intake (36.77 ± 1.34 vs. 46.81 ± 1.36, *P* = 0.011). This association remained statistically significant after adjusting for age and gender (F (2, 90) = 5.035, P = 0.008), and energy intake (F (2, 89) = 3.58, P = 0.032), but not after Bonferroni adjustment (*P*>0.002).

No significant difference between tertiles of protein intake was observed in plasma NAD^+^/NADH ratio (F (2, 91) = 2.80, *P* = 0.066) (Fig 1), NADP^+^ (F (2, 92) = 1.08, *P* = 0.344), cADPR (F (2, 92) = 0.92, *P* = 0.920), NAM (F (2, 89) = 0.99, *P* = 0.991) and MeNAM (F (2, 89) = 0.99, *P* = 0.991).

### Association between protein intake and plasma levels of urea

A statistically significant difference in plasma urea levels was observed between protein intake tertiles (F (2, 95) = 6.97, *P*≤0.001). A Tukey post hoc test revealed that plasma urea levels were significantly higher in the third tertile compared to the first tertile of protein intake (6.15 ± 1.51 vs. 4.83 ± 1.21, *P*≤0.001). This association remained statistically significant after adjusting for age, gender, eGFR and energy intake (F (2, 90) = 9.84, *P*≤0.001), and also after Bonferroni adjustment (P≤0.002).

### Association between plasma levels of urea, NAD^+^ and NAD^+^ metabolites

**Fig 2:**
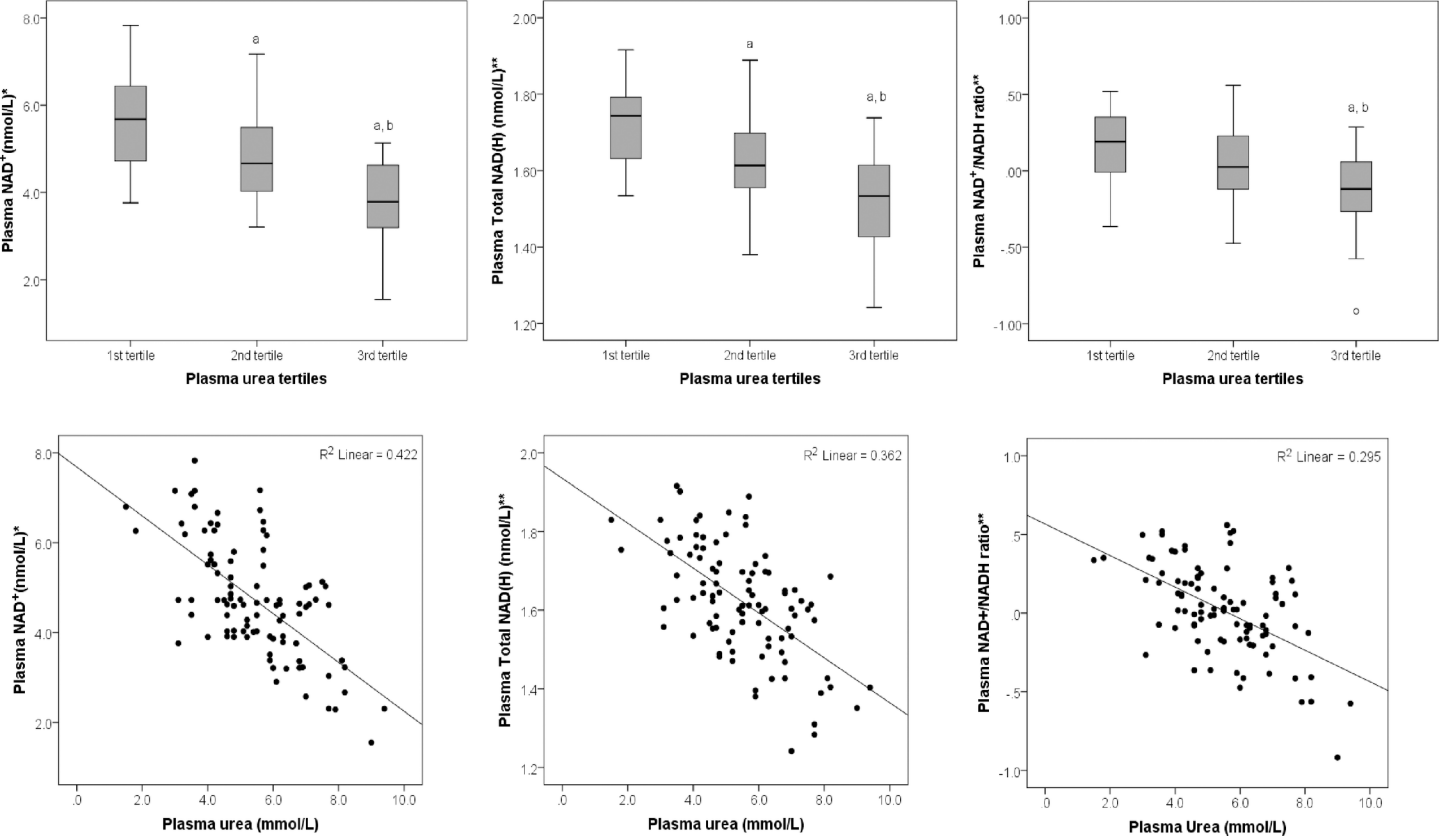
Mean plasma levels of NAD^+^, Total NAD(H), and NAD^+^/NADH ratio across tertiles of plasma urea (on the left), and corresponding linear graphs for the association between levels of plasma urea, and NAD^+^, Total NAD(H) and NAD^+^/NADH ratio (on the right). *Data represented as square root NAD^+^, **Data represented as base-10 log-transformed Total NAD^+^ and NADH, and NAD^+^/NADH ratio. a significantly different compared to 1^st^ tertile, b significantly different compared to the 2^nd^ tertile.

A statistically significant difference in plasma NAD^+^ levels was observed between plasma urea tertiles (F (2, 93) = 25.11, *P*≤0.001) (Fig 2). This association remained statistically significant after adjusting for age, gender, eGFR and energy intake (F (2, 87) = 18.72, *P*≤0.001), and also after Bonferroni adjustment (P≤0.002).

No significant difference in plasma NADH levels was observed between plasma urea tertiles (F (2, 92) = 1.28, *P* = 0.284).

There was a statistically significant difference in plasma Total NAD(H) levels between plasma urea tertiles (F (2, 93) = 21.10, *P*≤0.001) (Fig 2). This association remained statistically significant after adjusting for age, gender, eGFR and energy intake (F (2, 87) = 12.97, *P*≤0.001), and also after Bonferroni adjustment (P≤0.002).

A statistically significant difference in plasma NAD^+^/NADH ratio was observed between plasma urea tertiles (F (2, 92) = 12.97, *P*≤0.001) (Fig 2). This association remained statistically significant after adjusting for age, gender, eGFR and energy intake (F (2, 86) = 11.20, *P*≤0.001), and also after Bonferroni adjustment (P≤0.002).

No significant difference between plasma urea tertiles was observed in plasma NADP^+^ (F (2, 93) = 0.52, *P* = 0.599), cADPR (F (2, 93) = 0.21, *P* = 0.814), NAM (F (2, 90) = 0.97, *P* = 0.384) and MeNAM (F (2, 90) = 0.97, *P* = 0.384).

### Association between plasma levels of urea and inflammation

**Fig 3:**
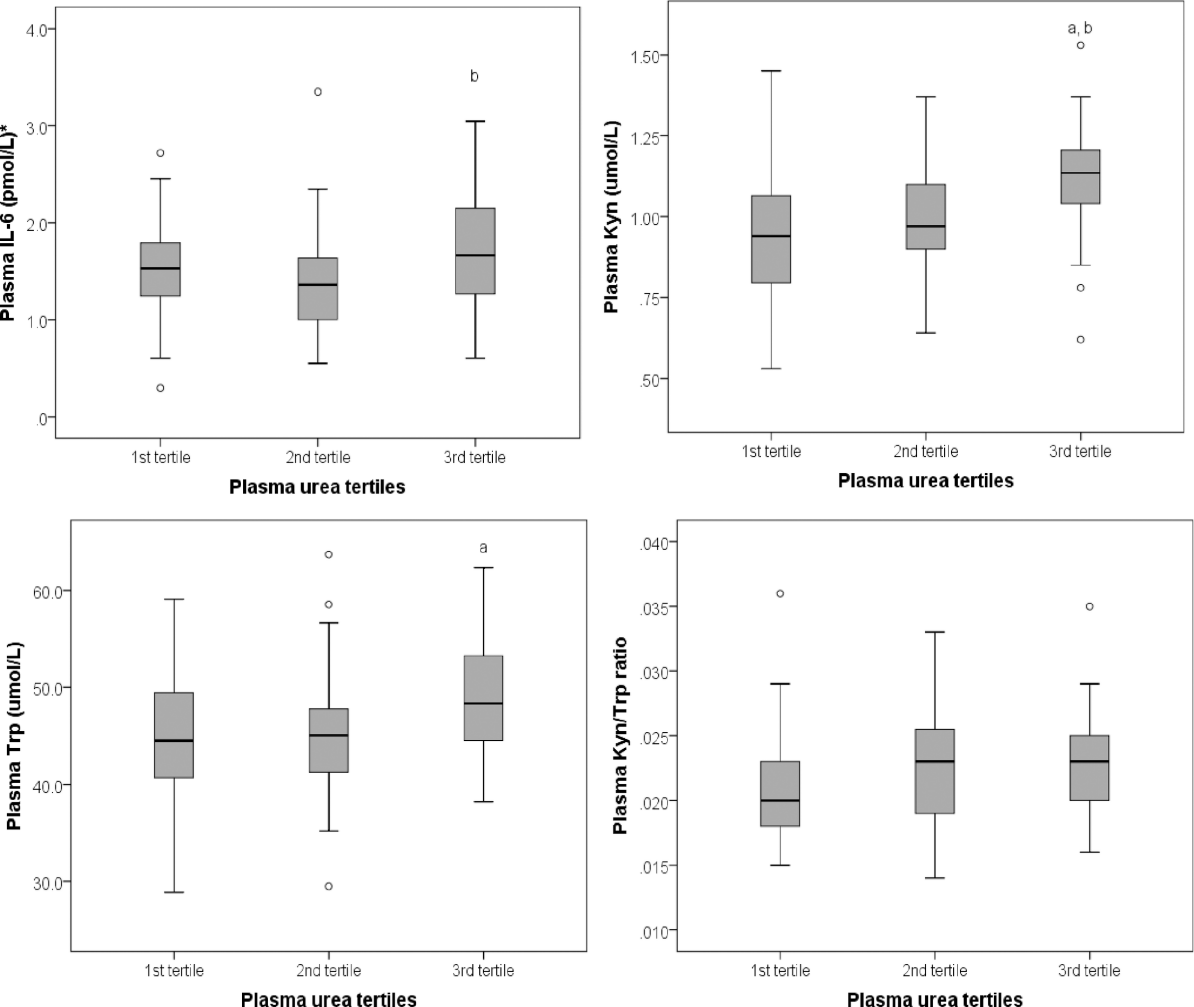
Mean plasma levels of interleukin-6 (IL-6), Kynurenine (Kyn), Tryptophan (Trp) and Kyn/Trp ratio across tertiles of plasma urea. *Data represented as square root IL-6. *a* significantly different compared to 1^st^ tertile, *b* significantly different compared to the 2^nd^ tertile.

A statistically significant difference in plasma IL-6 levels was observed between tertiles of plasma urea (F (2, 94) = 3.30, *P* = 0.041) (Fig 3). A Tukey post hoc test revealed that mean plasma IL-6 levels were significantly higher in the third tertile compared to the second tertile (3.00 ± 0.32 vs. 1.94 ± 0.33, *P* = 0.036) of plasma urea. However, this association did not remain statistically significant after adjusting for age, gender, eGFR and energy intake (F (2, 88) = 4.39, *P* = 0.090), and also after Bonferroni adjustment (P>0.002).

There was a statistically significant difference in plasma Trp levels between tertiles of plasma urea (F (2, 96) = 4.23, *P* = 0.017) (Fig 3). A Tukey post hoc test revealed that mean plasma Trp levels were significantly higher in the third tertile (49.09 ± 6.58) compared to the first tertile (44.28 ± 8.07) of plasma urea. This association remained significant after adjusting for age, gender, eGFR and energy intake (F (2, 90) = 3.74, *P* = 0.028), but not after Bonferroni adjustment (P>0.002).

Also, there was a statistically significant difference in plasma Kyn levels between tertiles of plasma urea (F (2, 95) = 7.41, *P*≤0.001) (Fig 3). A Tukey post hoc test revealed that mean plasma Kyn levels were significantly higher in the third tertile (1.11 ± 0.18) compared to both the second tertile (0.98 ± 0.19, *P* = 0.017) and first tertile (0.93 ± 0.21, *P*≤0.001) of plasma urea. This association remained significant after adjusting for age and gender (F (2, 93) = 4.46, *P* = 0.014), and also after Bonferroni adjustment (P≤0.002). However, it did not remain statistically significant after further adjustment for eGFR and energy intake (F (2, 89) = 1.30, *P* = 0.278).

No significant difference in plasma Kyn/Trp ratio levels was observed between plasma urea tertiles (F (2, 95) = 1.15, *P* = 0.322) (Fig 3).

## Discussion

Protein restriction, as a promising strategy to delay ageing, has gained considerable support from both animal and human studies in recent years [7, 8, 9]. While the effect of protein and amino acid restriction on levels and/or activity of longevity associated molecules have been previously investigated [9, 10, 11], the mechanism linking protein restriction to longevity is still not completely understood.

Nicotinamide adenine dinucleotide (NAD^+^), long recognised for its essential role in energy production and hundreds of redox reactions [12], has recently become a molecule of interest due to its central role in a range of ageing related pathways [13–18]. In addition to its well-known role in oxidative phosphorylation and ATP production, NAD^+^ is also a crucial substrate for three groups of enzymes involved in DNA repair, immune modulation and epigenetic signalling, including: (i) poly ADP-ribose polymerases (PARPs) [13]; (ii) CD38 glycohydrolase or cyclic ADP-ribose synthase [14, 15]; and (iii) sirtuin deacetylases (SIRTs) [16]. More importantly, NAD^+^ levels have been shown to decline with age [19– 21] highlighting its likely importance in ageing processes. We therefore questioned whether a link exists between protein intake and plasma NAD^+^ levels that may affect the ageing process.

Data from this study showed for the first time, a significant decrease in plasma NAD^+^ and Total NAD(H) levels with increasing protein intake, which was independent of age, gender and even energy intake. However, we did not observe any significant associations between protein intake and plasma NADH, NAD^+^/NADH ratios and NADP^+^. In support of this finding, the hepatic tissue NAD^+^ content of rats fed excess dietary leucine has previously been reported to be significantly lower than control rats, while no significant changes in NADH and NADP^+^ levels were observed. This decrease in NAD^+^ levels was reported to be independent of the alteration in NAD^+^ metabolism [28].

To corroborate these observations, we then looked for possible associations between plasma NAD^+^ and Total NAD(H) levels, and plasma levels of urea, the main end product of amino acid catabolism. As expected, urea levels were positively associated with protein intake as reported by others [29]. In addition, we observed a robust inverse association between plasma urea levels and NAD^+^ and Total NAD(H) concentrations that was independent of age, gender, eGFR, and energy intake. This finding is novel and suggests a link between protein metabolism and plasma NAD^+^ levels.

**Fig 4:**
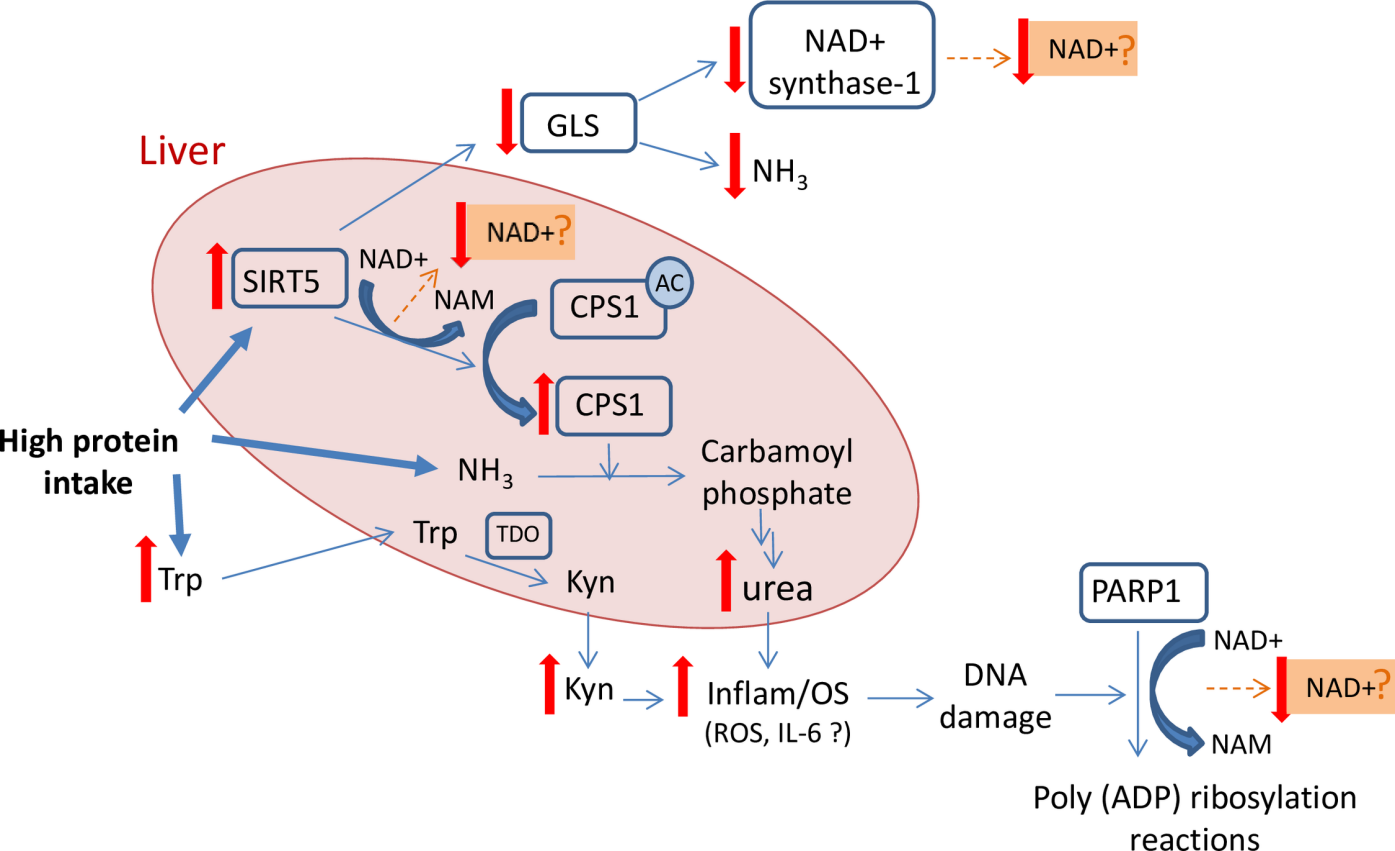
Theoretical mechanism for the contribution of high protein intake to NAD^+^ and its metabolites levels [30– 34, 44, 48]. SIRT5: Sirtuin 5; NAD^+^: Nicotinamide adenine dinucleotide; NAM: Nicotinamidec; NH_3_: Ammonia; GLS: Glutaminase; AC: Acetyl; CPS1: Carbamoyl phosphate synthetase 1; Trp: Tryptophan; Kyn: Kynurenine; TDO: Tryptophan 2,3-Dioxygenase; Inflam: inflammation; OS: Oxidative stress; ROS: Reactive oxygen specious; Interleukin-6; cADPR: Cyclic ADP ribose; PARPs: Poly ADP-ribose polymerases.

While mechanisms responsible for these observations are not completely understood, and our observational data limit definitive mechanistic conclusions, plausible hypotheses are suggested that may assist future investigations. It has been shown that among the seven known NAD^+^-dependant SIRTs, the mitochondrial enzyme SIRT5 plays a major role in protein metabolism in animals. It has been observed by others that during a high protein diet, SIRT5 is activated triggering the de-acetylation and up-regulation of carbamoyl phosphate synthetase 1 (CPS1) activity, a rate-limiting enzyme of the urea cycle in liver [30, 31]. The urea cycle is responsible for disposing of the toxic ammonia produced in protein catabolism by converting it to urea [31], and by up-regulating CPS1 activity, SIRT5 can play a pivotal role in ammonia detoxification (Fig 4). As SIRT5 is an NAD^+^-dependant enzyme, significant amounts of NAD^+^ can be consumed during this process which could conceivably contribute to the reduced plasma NAD^+^ levels. However, this hypothesis requires verification. Furthermore, SIRT5 has also been shown to inhibit glutaminase (GLS), an enzyme responsible for the conversion of glutamine to glutamate (with release of ammonia), in human and animal cell cultures in order to control plasma ammonia levels [32]. As GLS also supplies the ammonia group for the last step of NAD^+^ biosynthesis that is catalysed by NAD^+^ synthetase-1 [33], its inhibition by SIRT5 suggests another potential contributor to a urea-associated reduction in plasma NAD^+^ levels (Fig 4). Again a hypothesis that requires further investigation. Finally, urea treatment (20mM) in human endothelial cells has recently been shown to induce mitochondrial production of excess reactive oxygen species (ROS). The urea-induced ROS has been shown to induce DNA damage and consequently increase the activity of the NAD^+^-dependant PARP-1 to repair the damaged DNA [34]. As with SIRT5, the over-activation of PARP-1 can lead to accelerated consumption of NAD^+^ molecules with potential for the consequent reduction in plasma NAD^+^ levels (Fig 4). However, as the range of urea levels in this study were within normal limits (<10mM) it will be important to validate whether this mechanism is in fact operational in vivo within the normal range.

While the data presented in this report is consistent with the above hypotheses we were somewhat surprised to find no significant association between plasma levels of urea or protein intake and the NAD^+^ metabolites NAM or MeNAM. NAM is an NAD^+^ precursor and also an NAD^+^ metabolite produced when the NAD^+^ molecule is catabolised by any of the NAD^+^-dependant enzymes such as PARPs, CD38 glycohydrolases or SIRTs [14, 25, 35]. Under normal conditions NAM can be reconverted to NAD^+^ via the salvage pathway [14]. However, when NAD^+^ is consumed at a rate greater than it can be synthetised plasma NAD^+^ levels are reduced with a concomitant rise in NAM levels [13, 25]. Consequently, NAM can be further metabolized to MeNAM and excreted via urine [25]. Thus, the lack of association between protein intake or urea levels and NAM or meNAM may be due to a significant loss of NAM and MeNAM in the urine which was unaccounted for in the study design [36, 37] as a 24 hour urine sample collection was logistically untenable for this cohort.

We did not observe any significant differences in plasma NADH levels between urea tertiles. This is consistent with a previous report in which decreases in hepatic NAD^+^ but not NADH was observed in an animal model fed a diet high in leucine [28]. However, we did observe that plasma NAD^+^/ NADH ratio levels decrease as plasma urea increases. The NAD^+^/ NADH ratio is a recognised marker of intracellular metabolic and redox status [19, 35]. A decrease in the NAD^+^/NADH ratio indicates a shift in redox balance toward a more reduced state, a situation that occurs as a result of cellular oxidative stress, excess energy intake or reduced mitochondrial oxidative phosphorylation [19, 38].

Analogous to our observations for NADH, no significant differences in plasma NADP^+^ levels were observed between urea tertiles. This was also consistent with data reported in the above cited animal study where NAD^+^ but not NADP^+^ was shown to decrease [28]. NADP is synthesised by the enzyme, NAD^+^-kinase from the two substrates, NAD^+^ and ATP. As with ATP, it would appear that NAD^+^ needs to decrease below a critical threshold before a decrease in NADP^+^ levels is observed [39].

Importantly, we did observe a urea-associated increase in the inflammatory cytokine IL-6. While no data appears to exist for this association in cohorts within the normal plasma urea range, previous researchers have observed high plasma IL-6 levels in uremic patients [40]. As increased inflammatory activity is a primary driver of oxidative damage [41], the elevated IL-6 levels seen in the higher urea tertiles in our study is consistent with the hypothesised urea-induced oxidative DNA damage and accelerated PARP-1 induced NAD^+^ turnover.

As observed by others [42], urea levels were also positively associated with plasma kynurenine (Kyn) in our cohort. Kyn is produced from tryptophan (Trp) via the Kynurenine pathway and its levels have been shown to significantly increase when the pathway’s rate limiting enzyme Indoleamine 2, 3 dioxygenase (IDO) is induced by a T-helper 1 (Th1) immune response [43, 44]. However, in our study, plasma Trp levels were also positively and independently associated with plasma urea levels. The observed association between urea, Kyn and Trp levels, together with no association between urea and Kyn/Trp ratio in our study, suggest that the increase in Kyn levels in higher urea tertiles is not due to a urea-induced Th1 immune response, but is simply due to the increase in tryptophan, derived from a higher intake of dietary protein. As Th1 cytokines promote differentiation of monocytes and increase expression of CD38 [45], this apparent lack of Th1 induction is also consistent with our observation of no association between plasma levels of urea or protein intake and the CD38 metabolite cADPR [46]. The metabolite cADPR is produced when NAD^+^ is metabolised by CD38 glycohydrolase [14] and therefore this lack of association between plasma urea or protein intake and cADPR suggests no change in this enzyme’s activity [47].

However, the observed increase in IL-6 levels in higher urea tertiles in our study is consistent with an elevated (though subclinical) inflammatory process at higher urea levels. Importantly, Kyn itself has been shown to exhibit pro-oxidant effects by producing ROS such as the superoxide radical [44, 48]. Taken together, our observation that elevated urea levels are associated with increased plasma IL-6 and Kyn levels is consistent with some level of urea-induced inflammatory activity (though not likely Th-1 mediated) and potential for oxidative damage [34].

## Limitations

While we believe that the observations reported in this study are statistically valid, and informative, we acknowledge that some limitations exist. First, the cross-sectional design of this study does not allow for the confirmation of causality, but it can generate hypotheses and thus stimulate future research. Second, the relatively small number of subjects may reduce the sensitivity for identification of relationships with small effect sizes so that some relationships may have gone unnoticed. Furthermore, because protein intake information was self-reported, increased measurement error may occur. However, the use of a well validated questionnaire should have decreased this potential error [22]. Also, our findings rely on plasma (not urine) values of urea and NAD^+^ metabolites, with no measurement of plasma/urea levels of ammonia or the enzyme SIRT5 activity. Therefore, in order to establish the mechanisms behind the observed association between protein metabolism and NAD^+^, future longitudinal studies overcoming these limitations are required. In particular studies designed to assess changes in hepatic activity or expression of the enzymes SIRT5, NAD^+^ synthase and PARP1, and tissue/plasma levels of ammonia in addition to oxidative and inflammatory markers in animals fed diets containing different protein percentages are suggested.

## Conclusion

This study has reported, for the first time, novel associations between both protein intake and the amino acid metabolite urea, and the ubiquitous nucleotide, NAD^+^ in a cohort of healthy middle-aged human subjects. These observations may be relevant in developing a model that explains how protein restriction is affective in promoting longevity as observed in several studies. As the activity of the NAD^+^ dependant enzymes of SIRT5 and PARP1 have recently been reported to increase in response to a chronic high protein diet and urea treatment, respectively [30, 34] it is not unreasonable to suggest that the consumption of NAD^+^ by these enzymes may play a role in the observed decrease in plasma NAD^+^ levels at higher protein and urea levels. However, further prospective studies are required to confirm this hypothesis.

## Supporting information

S1 Fig Chromatograms of NAD+, NADH and NADP+ measured by liquid chromatography coupled to tandem mass spectrometry (LC/MS/MS) technique. (TIF)

## Abbreviations

NAD^+^: Nicotinamide adenine dinucleotide
PARPs: Poly ADP-ribose polymerases
SIRTs: Sirtuins
CD38: Cluster of differentiation 38
eGFR: Estimated Glomerular filtration rate
cADPR: Cyclic ADP ribose
NAM: Nicotinamide
MeNAM: N-methylnicotinamide
IL-6: Interleukin-6
Kyn: Kynurenine
Trp: Tryptophan
CPS1: Carbamoyl phosphate synthetase 1
GLS: Glutaminase
