# Supplementary figures and images for: High protein intake is associated with low plasma NAD+ levels in a healthy human cohort
Source: PLoS One. 2018 Aug 16;13(8):e0201968. doi: 10.1371/journal.pone.0201968 (PMC6095538; doi:10.1371/journal.pone.0201968)

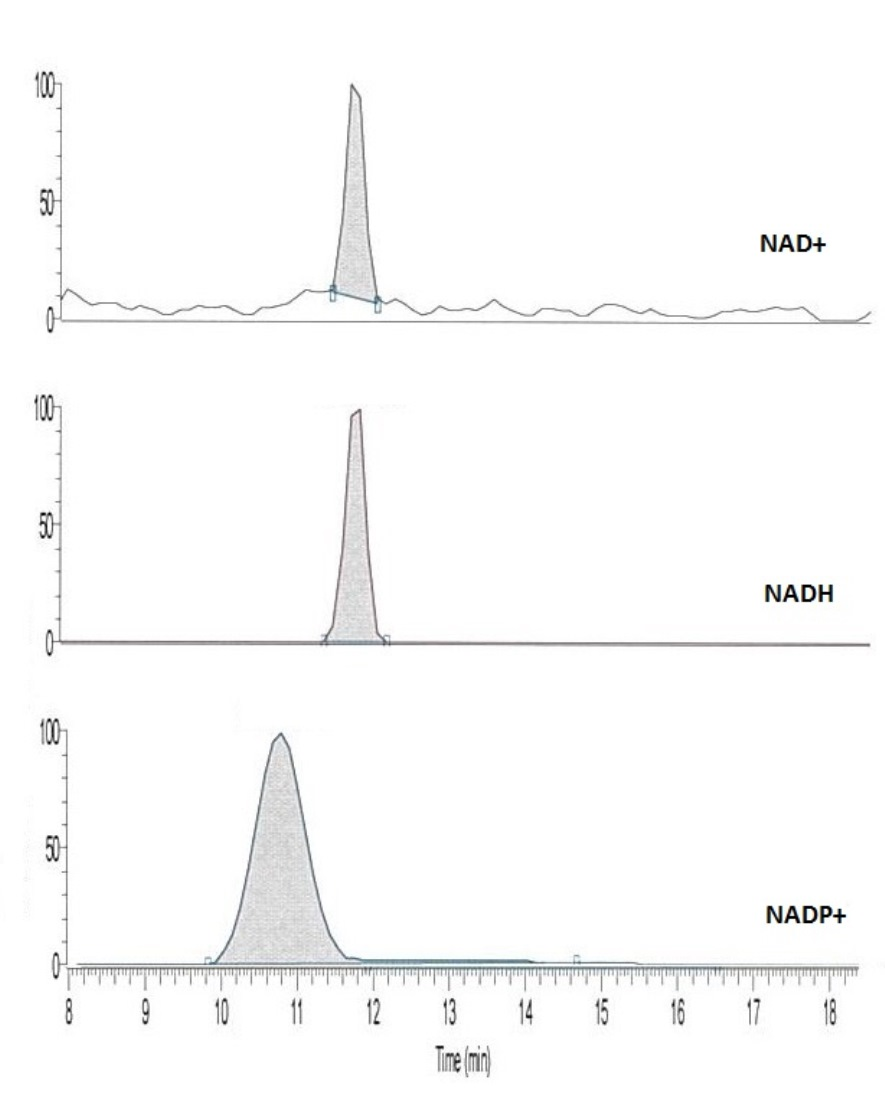

Supplement: S1 Fig — (TIF) [file pone.0201968.s001.tif]
